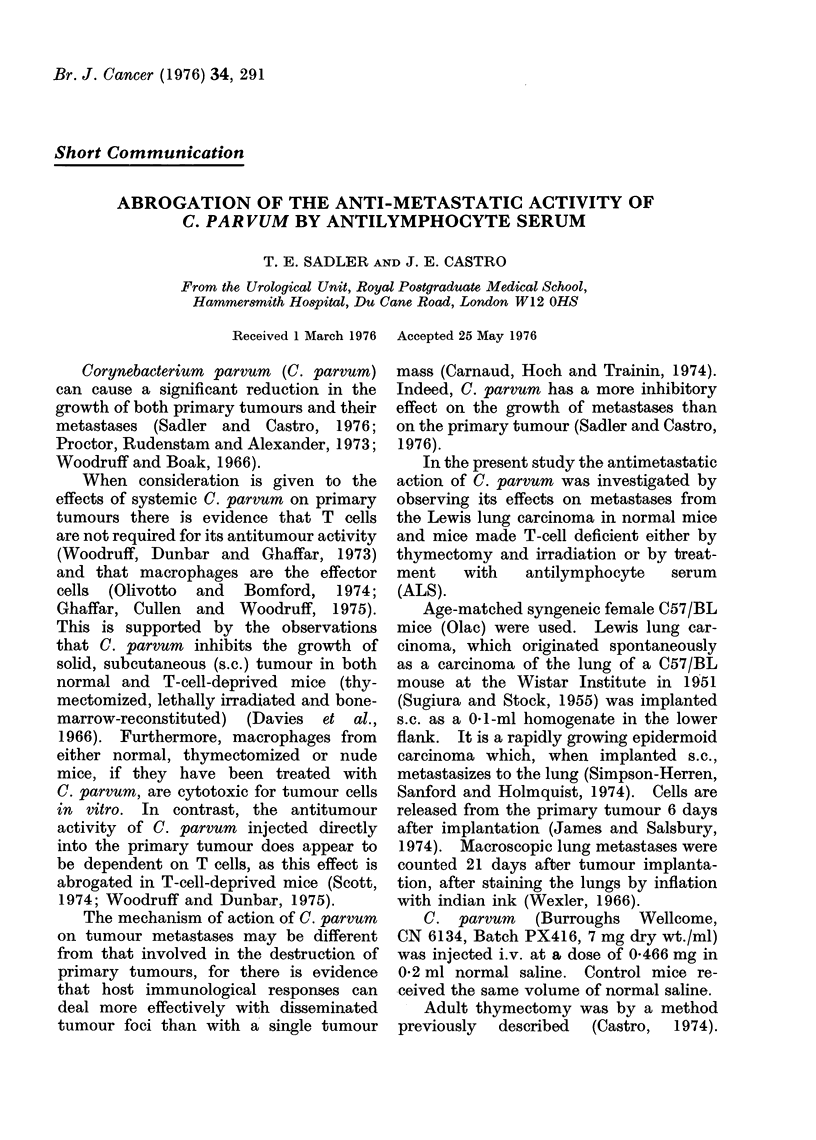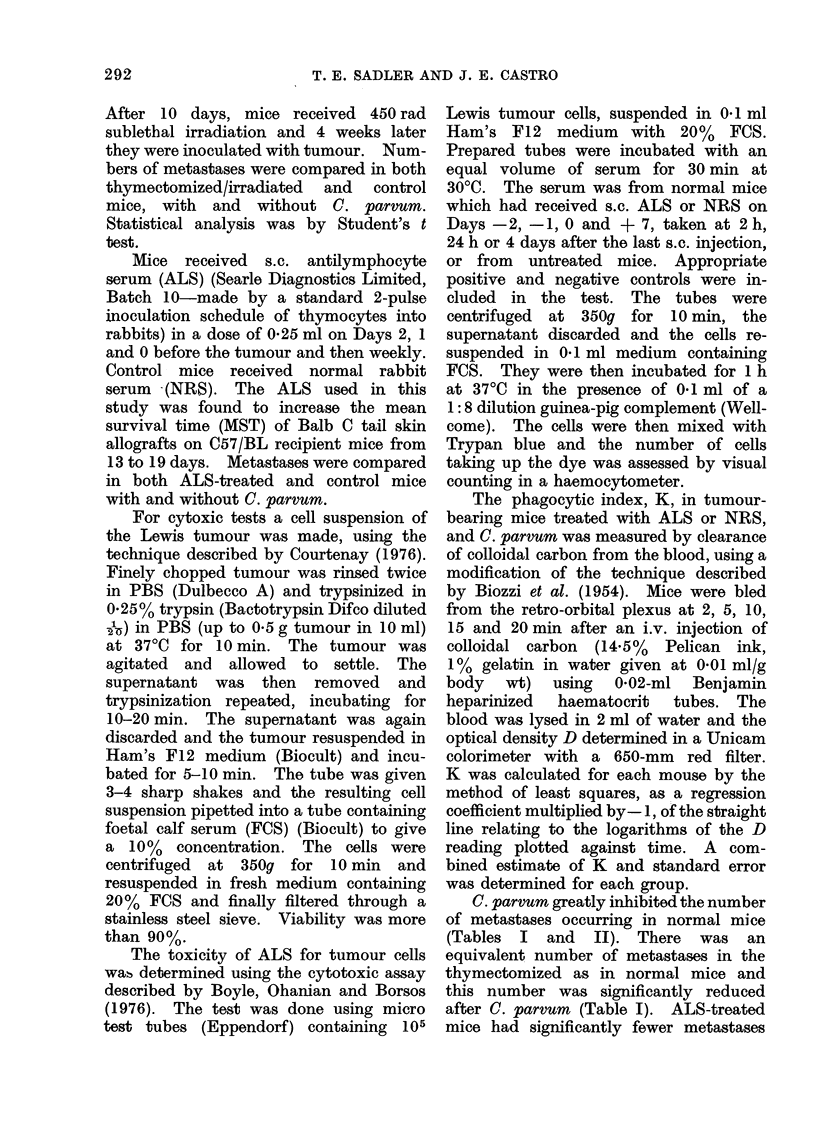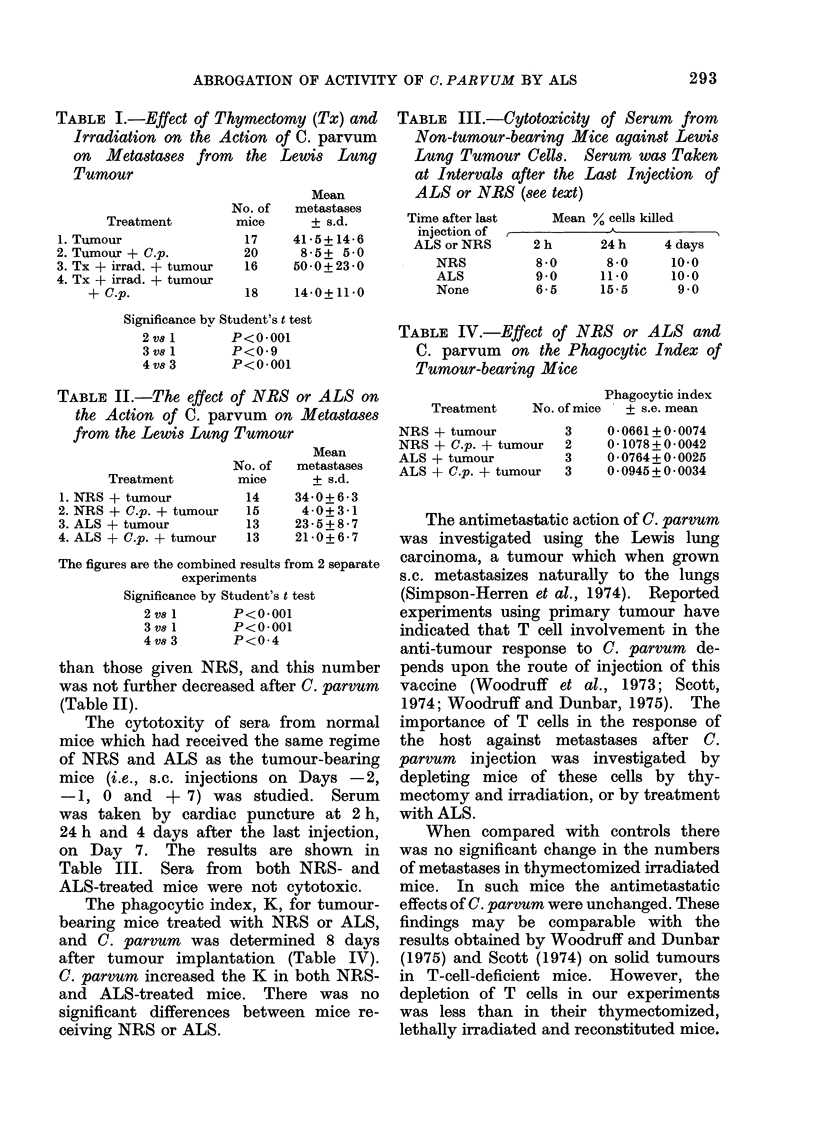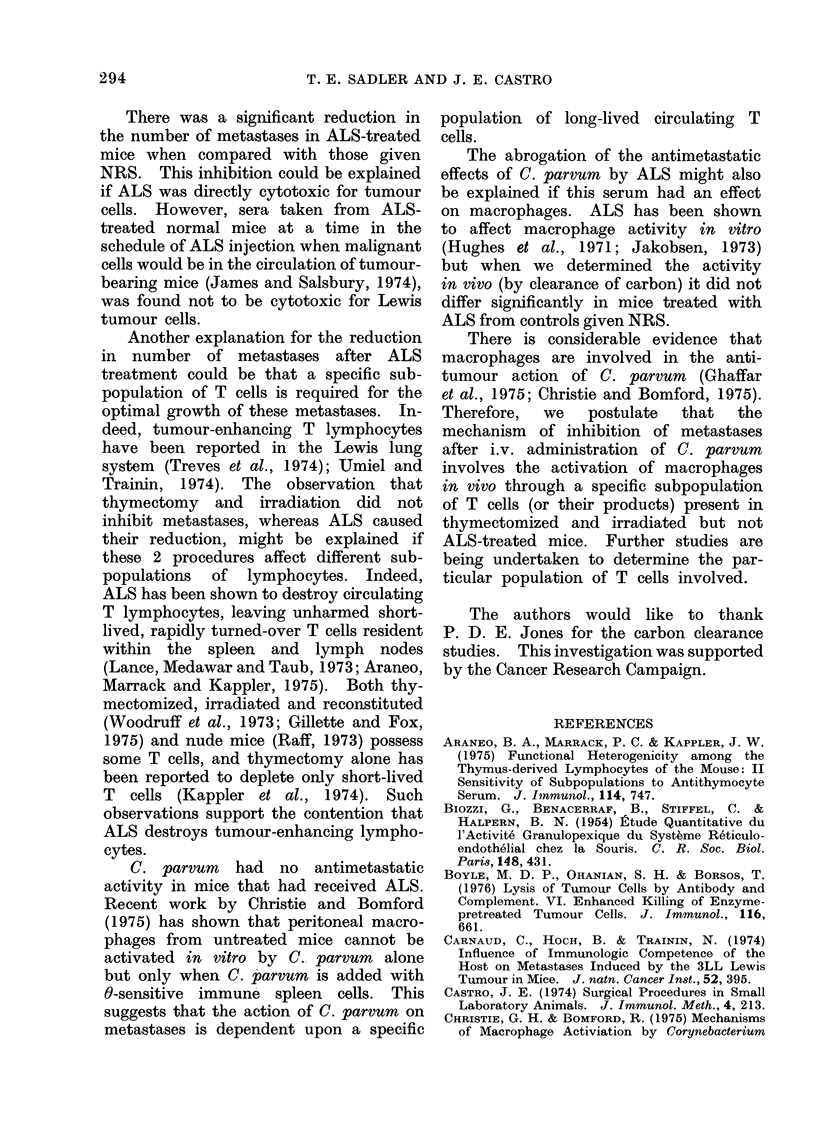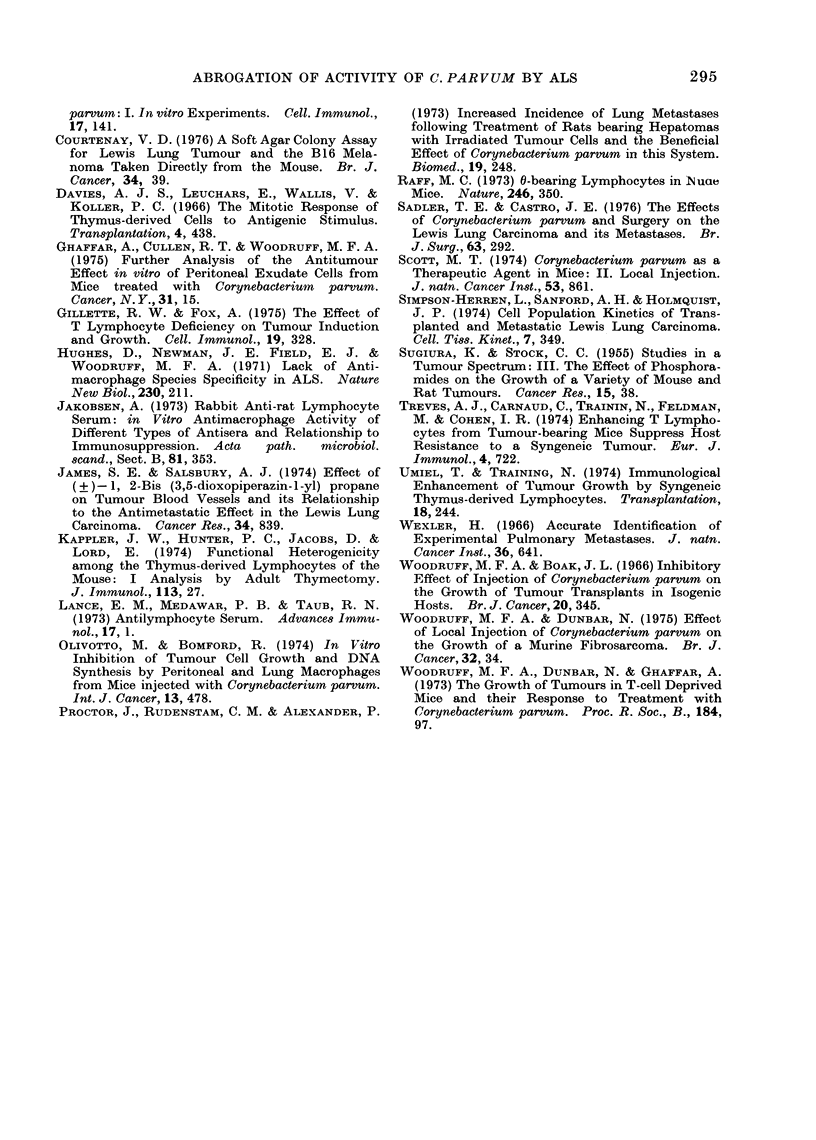# Abrogation of the anti-metastatic activity of C. parvum by antilymphocyte serum.

**DOI:** 10.1038/bjc.1976.165

**Published:** 1976-09

**Authors:** T. E. Sadler, J. E. Castro


*Br. J. Cancer* (1976) **34**, 291

***Short Communication***

### ABROGATION OF THE ANTI-METASTATIC ACTIVITY OF *C. PARVUM* BY ANTILYMPHOCYTE SERUM

T. E. SADLER AND J. E. CASTRO

*From the Urological Unit, Royal Postgraduate Medical School, Hammersmith Hospital, Du Cane Road, London W12 0HS*

Received 1 March 1976 Accepted 25 May 1976

*Corynebacterium parvum* (*C. parvum*) can cause a significant reduction in the growth of both primary tumours and their metastases (Sadler and Castro, 1976; Proctor, Rudenstam and Alexander, 1973; Woodruff and Boak, 1966).

When consideration is given to the effects of systemic *C. parvum* on primary tumours there is evidence that T cells are not required for its antitumour activity (Woodruff, Dunbar and Ghaffar, 1973) and that macrophages are the effector cells (Olivotto and Bomford, 1974; Ghaffar, Cullen and Woodruff, 1975). This is supported by the observations that *C. parvum* inhibits the growth of solid, subcutaneous (s.c.) tumour in both normal and T-cell-deprived mice (thymectomized, lethally irradiated and bone-marrow-reconstituted) (Davies *et al.*, 1966). Furthermore, macrophages from either normal, thymectomized or nude mice, if they have been treated with *C. parvum*, are cytotoxic for tumour cells *in vitro*. In contrast, the antitumour activity of *C. parvum* injected directly into the primary tumour does appear to be dependent on T cells, as this effect is abrogated in T-cell-deprived mice (Scott, 1974; Woodruff and Dunbar, 1975).

The mechanism of action of *C. parvum* on tumour metastases may be different from that involved in the destruction of primary tumours, for there is evidence that host immunological responses can deal more effectively with disseminated tumour foci than with a single tumour mass (Carnaud, Hoch and Trainin, 1974). Indeed, *C. parvum* has a more inhibitory effect on the growth of metastases than on the primary tumour (Sadler and Castro, 1976).

In the present study the antimetastatic action of *C. parvum* was investigated by observing its effects on metastases from the Lewis lung carcinoma in normal mice and mice made T-cell deficient either by thymectomy and irradiation or by treatment with antilymphocyte serum (ALS).

Age-matched syngeneic female C57/BL mice (Olac) were used. Lewis lung carcinoma, which originated spontaneously as a carcinoma of the lung of a C57/BL mouse at the Wistar Institute in 1951 (Sugiura and Stock, 1955) was implanted s.c. as a 0·1-ml homogenate in the lower flank. It is a rapidly growing epidermoid carcinoma which, when implanted s.c., metastasizes to the lung (Simpson-Herren, Sanford and Holmquist, 1974). Cells are released from the primary tumour 6 days after implantation (James and Salsbury, 1974). Macroscopic lung metastases were counted 21 days after tumour implantation, after staining the lungs by inflation with indian ink (Wexler, 1966).

*C. parvum* (Burroughs Wellcome, CN 6134, Batch PX416, 7 mg dry wt./ml) was injected i.v. at a dose of 0·466 mg in 0·2 ml normal saline. Control mice received the same volume of normal saline.

Adult thymectomy was by a method previously described (Castro, 1974).

After 10 days, mice received 450 rad sublethal irradiation and 4 weeks later they were inoculated with tumour. Numbers of metastases were compared in both thymectomized/irradiated and control mice, with and without *C. parvum*. Statistical analysis was by Student's *t* test.

Mice received s.c. antilymphocyte serum (ALS) (Searle Diagnostics Limited, Batch 10—made by a standard 2-pulse inoculation schedule of thymocytes into rabbits) in a dose of 0·25 ml on Days 2, 1 and 0 before the tumour and then weekly. Control mice received normal rabbit serum (NRS). The ALS used in this study was found to increase the mean survival time (MST) of Balb C tail skin allografts on C57/BL recipient mice from 13 to 19 days. Metastases were compared in both ALS-treated and control mice with and without *C. parvum.*

For cytoxic tests a cell suspension of the Lewis tumour was made, using the technique described by Courtenay (1976). Finely chopped tumour was rinsed twice in PBS (Dulbecco A) and trypsinized in 0·25% trypsin (Bactotrypsin Difco diluted $\frac{1}{20}$) in PBS (up to 0·5 g tumour in 10 ml) at 37°C for 10 min. The tumour was agitated and allowed to settle. The supernatant was then removed and trypsinization repeated, incubating for 10–20 min. The supernatant was again discarded and the tumour resuspended in Ham's F12 medium (Biocult) and incubated for 5–10 min. The tube was given 3–4 sharp shakes and the resulting cell suspension pipetted into a tube containing foetal calf serum (FCS) (Biocult) to give a 10% concentration. The cells were centrifuged at 350*g* for 10 min and resuspended in fresh medium containing 20% FCS and finally filtered through a stainless steel sieve. Viability was more than 90%.

The toxicity of ALS for tumour cells was determined using the cytotoxic assay described by Boyle, Ohanian and Borsos (1976). The test was done using micro test tubes (Eppendorf) containing $10^5$ Lewis tumour cells, suspended in 0·1 ml Ham's F12 medium with 20% FCS. Prepared tubes were incubated with an equal volume of serum for 30 min at 30°C. The serum was from normal mice which had received s.c. ALS or NRS on Days −2, −1, 0 and + 7, taken at 2 h, 24 h or 4 days after the last s.c. injection, or from untreated mice. Appropriate positive and negative controls were included in the test. The tubes were centrifuged at 350*g* for 10 min, the supernatant discarded and the cells resuspended in 0·1 ml medium containing FCS. They were then incubated for 1 h at 37°C in the presence of 0·1 ml of a 1:8 dilution guinea-pig complement (Wellcome). The cells were then mixed with Trypan blue and the number of cells taking up the dye was assessed by visual counting in a haemocytometer.

The phagocytic index, K, in tumour-bearing mice treated with ALS or NRS, and *C. parvum* was measured by clearance of colloidal carbon from the blood, using a modification of the technique described by Biozzi *et al.* (1954). Mice were bled from the retro-orbital plexus at 2, 5, 10, 15 and 20 min after an i.v. injection of colloidal carbon (14·5% Pelican ink, 1% gelatin in water given at 0·01 ml/g body wt) using 0·02-ml Benjamin heparinized haematocrit tubes. The blood was lysed in 2 ml of water and the optical density *D* determined in a Unicam colorimeter with a 650-mm red filter. K was calculated for each mouse by the method of least squares, as a regression coefficient multiplied by −1, of the straight line relating to the logarithms of the *D* reading plotted against time. A combined estimate of K and standard error was determined for each group.

*C. parvum* greatly inhibited the number of metastases occurring in normal mice (Tables I and II). There was an equivalent number of metastases in the thymectomized as in normal mice and this number was significantly reduced after *C. parvum* (Table I). ALS-treated mice had significantly fewer metastases

TABLE I.—*Effect of Thymectomy (Tx) and Irradiation on the Action of* C. parvum *on Metastases from the Lewis Lung Tumour*

| Treatment | No. of mice | Mean metastases ± s.d. |
|---|---|---|
| 1. Tumour | 17 | 41·5 ± 14·6 |
| 2. Tumour + *C.p.* | 20 | 8·5 ± 5·0 |
| 3. Tx + irrad. + tumour | 16 | 50·0 ± 23·0 |
| 4. Tx + irrad. + tumour + *C.p.* | 18 | 14·0 ± 11·0 |

Significance by Student's *t* test

| | |
|---|---|
| 2 *vs* 1 | $P < 0·001$ |
| 3 *vs* 1 | $P < 0·9$ |
| 4 *vs* 3 | $P < 0·001$ |

TABLE II.—*The effect of NRS or ALS on the Action of* C. parvum *on Metastases from the Lewis Lung Tumour*

| Treatment | No. of mice | Mean metastases ± s.d. |
|---|---|---|
| 1. NRS + tumour | 14 | 34·0 ± 6·3 |
| 2. NRS + *C.p.* + tumour | 15 | 4·0 ± 3·1 |
| 3. ALS + tumour | 13 | 23·5 ± 8·7 |
| 4. ALS + *C.p.* + tumour | 13 | 21·0 ± 6·7 |

The figures are the combined results from 2 separate experiments

Significance by Student's *t* test

| | |
|---|---|
| 2 *vs* 1 | $P < 0·001$ |
| 3 *vs* 1 | $P < 0·001$ |
| 4 *vs* 3 | $P < 0·4$ |

than those given NRS, and this number was not further decreased after *C. parvum* (Table II).

The cytotoxity of sera from normal mice which had received the same regime of NRS and ALS as the tumour-bearing mice (*i.e.*, s.c. injections on Days −2, −1, 0 and + 7) was studied. Serum was taken by cardiac puncture at 2 h, 24 h and 4 days after the last injection, on Day 7. The results are shown in Table III. Sera from both NRS- and ALS-treated mice were not cytotoxic.

The phagocytic index, K, for tumour-bearing mice treated with NRS or ALS, and *C. parvum* was determined 8 days after tumour implantation (Table IV). *C. parvum* increased the K in both NRS- and ALS-treated mice. There was no significant differences between mice receiving NRS or ALS.

TABLE III.—*Cytotoxicity of Serum from Non-tumour-bearing Mice against Lewis Lung Tumour Cells. Serum was Taken at Intervals after the Last Injection of ALS or NRS (see text)*

| Time after last injection of ALS or NRS | Mean % cells killed | | |
|---|---|---|---|
| | 2 h | 24 h | 4 days |
| NRS | 8·0 | 8·0 | 10·0 |
| ALS | 9·0 | 11·0 | 10·0 |
| None | 6·5 | 15·5 | 9·0 |

TABLE IV.—*Effect of NRS or ALS and* C. parvum *on the Phagocytic Index of Tumour-bearing Mice*

| Treatment | No. of mice | Phagocytic index ± s.e. mean |
|---|---|---|
| NRS + tumour | 3 | 0·0661 ± 0·0074 |
| NRS + *C.p.* + tumour | 2 | 0·1078 ± 0·0042 |
| ALS + tumour | 3 | 0·0764 ± 0·0025 |
| ALS + *C.p.* + tumour | 3 | 0·0945 ± 0·0034 |

The antimetastatic action of *C. parvum* was investigated using the Lewis lung carcinoma, a tumour which when grown s.c. metastasizes naturally to the lungs (Simpson-Herren *et al.*, 1974). Reported experiments using primary tumour have indicated that T cell involvement in the anti-tumour response to *C. parvum* depends upon the route of injection of this vaccine (Woodruff *et al.*, 1973; Scott, 1974; Woodruff and Dunbar, 1975). The importance of T cells in the response of the host against metastases after *C. parvum* injection was investigated by depleting mice of these cells by thymectomy and irradiation, or by treatment with ALS.

When compared with controls there was no significant change in the numbers of metastases in thymectomized irradiated mice. In such mice the antimetastatic effects of *C. parvum* were unchanged. These findings may be comparable with the results obtained by Woodruff and Dunbar (1975) and Scott (1974) on solid tumours in T-cell-deficient mice. However, the depletion of T cells in our experiments was less than in their thymectomized, lethally irradiated and reconstituted mice.

There was a significant reduction in the number of metastases in ALS-treated mice when compared with those given NRS. This inhibition could be explained if ALS was directly cytotoxic for tumour cells. However, sera taken from ALS-treated normal mice at a time in the schedule of ALS injection when malignant cells would be in the circulation of tumour-bearing mice (James and Salsbury, 1974), was found not to be cytotoxic for Lewis tumour cells.

Another explanation for the reduction in number of metastases after ALS treatment could be that a specific sub-population of T cells is required for the optimal growth of these metastases. Indeed, tumour-enhancing T lymphocytes have been reported in the Lewis lung system (Treves *et al.*, 1974); Umiel and Trainin, 1974). The observation that thymectomy and irradiation did not inhibit metastases, whereas ALS caused their reduction, might be explained if these 2 procedures affect different sub-populations of lymphocytes. Indeed, ALS has been shown to destroy circulating T lymphocytes, leaving unharmed short-lived, rapidly turned-over T cells resident within the spleen and lymph nodes (Lance, Medawar and Taub, 1973; Araneo, Marrack and Kappler, 1975). Both thymectomized, irradiated and reconstituted (Woodruff *et al.*, 1973; Gillette and Fox, 1975) and nude mice (Raff, 1973) possess some T cells, and thymectomy alone has been reported to deplete only short-lived T cells (Kappler *et al.*, 1974). Such observations support the contention that ALS destroys tumour-enhancing lymphocytes.

*C. parvum* had no antimetastatic activity in mice that had received ALS. Recent work by Christie and Bomford (1975) has shown that peritoneal macrophages from untreated mice cannot be activated *in vitro* by *C. parvum* alone but only when *C. parvum* is added with $\theta$-sensitive immune spleen cells. This suggests that the action of *C. parvum* on metastases is dependent upon a specific population of long-lived circulating T cells.

The abrogation of the antimetastatic effects of *C. parvum* by ALS might also be explained if this serum had an effect on macrophages. ALS has been shown to affect macrophage activity *in vitro* (Hughes *et al.*, 1971; Jakobsen, 1973) but when we determined the activity *in vivo* (by clearance of carbon) it did not differ significantly in mice treated with ALS from controls given NRS.

There is considerable evidence that macrophages are involved in the anti-tumour action of *C. parvum* (Ghaffar *et al.*, 1975; Christie and Bomford, 1975). Therefore, we postulate that the mechanism of inhibition of metastases after i.v. administration of *C. parvum* involves the activation of macrophages *in vivo* through a specific subpopulation of T cells (or their products) present in thymectomized and irradiated but not ALS-treated mice. Further studies are being undertaken to determine the particular population of T cells involved.

The authors would like to thank P. D. E. Jones for the carbon clearance studies. This investigation was supported by the Cancer Research Campaign.

### REFERENCES

Araneo, B. A., Marrack, P. C. & Kappler, J. W. (1975) Functional Heterogenicity among the Thymus-derived Lymphocytes of the Mouse: II Sensitivity of Subpopulations to Antithymocyte Serum. *J. Immunol.*, **114**, 747.

Biozzi, G., Benacerraf, B., Stiffel, C. & Halpern, B. N. (1954) Étude Quantitative du l'Activité Granulopexique du Système Réticulo-endothélial chez la Souris. *C. R. Soc. Biol. Paris*, **148**, 431.

Boyle, M. D. P., Ohanian, S. H. & Borsos, T. (1976) Lysis of Tumour Cells by Antibody and Complement. VI. Enhanced Killing of Enzyme-pretreated Tumour Cells. *J. Immunol.*, **116**, 661.

Carnaud, C., Hoch, B. & Trainin, N. (1974) Influence of Immunologic Competence of the Host on Metastases Induced by the 3LL Lewis Tumour in Mice. *J. natn. Cancer Inst.*, **52**, 395.

Castro, J. E. (1974) Surgical Procedures in Small Laboratory Animals. *J. Immunol. Meth.*, **4**, 213.

Christie, G. H. & Bomford, R. (1975) Mechanisms of Macrophage Activiation by *Corynebacterium*

*parvum*: I. *In vitro* Experiments. *Cell. Immunol.*, **17**, 141.

COURTENAY, V. D. (1976) A Soft Agar Colony Assay for Lewis Lung Tumour and the B16 Melanoma Taken Directly from the Mouse. *Br. J. Cancer*, **34**, 39.

DAVIES, A. J. S., LEUCHARS, E., WALLIS, V. & KOLLER, P. C. (1966) The Mitotic Response of Thymus-derived Cells to Antigenic Stimulus. *Transplantation*, **4**, 438.

GHAFFAR, A., CULLEN, R. T. & WOODRUFF, M. F. A. (1975) Further Analysis of the Antitumour Effect *in vitro* of Peritoneal Exudate Cells from Mice treated with *Corynebacterium parvum*. *Cancer, N.Y.*, **31**, 15.

GILLETTE, R. W. & FOX, A. (1975) The Effect of T Lymphocyte Deficiency on Tumour Induction and Growth. *Cell. Immunol.*, **19**, 328.

HUGHES, D., NEWMAN, J. E. FIELD, E. J. & WOODRUFF, M. F. A. (1971) Lack of Anti-macrophage Species Specificity in ALS. *Nature New Biol.*, **230**, 211.

JAKOBSEN, A. (1973) Rabbit Anti-rat Lymphocyte Serum: *in Vitro* Antimacrophage Activity of Different Types of Antisera and Relationship to Immunosuppression. *Acta path. microbiol. scand.*, Sect. B, **81**, 353.

JAMES, S. E. & SALSBURY, A. J. (1974) Effect of (±)−1, 2-Bis (3,5-dioxopiperazin-1-yl) propane on Tumour Blood Vessels and its Relationship to the Antimetastatic Effect in the Lewis Lung Carcinoma. *Cancer Res.*, **34**, 839.

KAPPLER, J. W., HUNTER, P. C., JACOBS, D. & LORD, E. (1974) Functional Heterogenicity among the Thymus-derived Lymphocytes of the Mouse: I Analysis by Adult Thymectomy. *J. Immunol.*, **113**, 27.

LANCE, E. M., MEDAWAR, P. B. & TAUB, R. N. (1973) Antilymphocyte Serum. *Advances Immunol.*, **17**, 1.

OLIVOTTO, M. & BOMFORD, R. (1974) *In Vitro* Inhibition of Tumour Cell Growth and DNA Synthesis by Peritoneal and Lung Macrophages from Mice injected with *Corynebacterium parvum*. *Int. J. Cancer*, **13**, 478.

PROCTOR, J., RUDENSTAM, C. M. & ALEXANDER, P. (1973) Increased Incidence of Lung Metastases following Treatment of Rats bearing Hepatomas with Irradiated Tumour Cells and the Beneficial Effect of *Corynebacterium parvum* in this System. *Biomed.*, **19**, 248.

RAFF, M. C. (1973) θ-bearing Lymphocytes in Nude Mice. *Nature*, **246**, 350.

SADLER, T. E. & CASTRO, J. E. (1976) The Effects of *Corynebacterium parvum* and Surgery on the Lewis Lung Carcinoma and its Metastases. *Br. J. Surg.*, **63**, 292.

SCOTT, M. T. (1974) *Corynebacterium parvum* as a Therapeutic Agent in Mice: II. Local Injection. *J. natn. Cancer Inst.*, **53**, 861.

SIMPSON-HERREN, L., SANFORD, A. H. & HOLMQUIST, J. P. (1974) Cell Population Kinetics of Transplanted and Metastatic Lewis Lung Carcinoma. *Cell. Tiss. Kinet.*, **7**, 349.

SUGIURA, K. & STOCK, C. C. (1955) Studies in a Tumour Spectrum: III. The Effect of Phosphoramides on the Growth of a Variety of Mouse and Rat Tumours. *Cancer Res.*, **15**, 38.

TREVES, A. J., CARNAUD, C., TRAININ, N., FELDMAN, M. & COHEN, I. R. (1974) Enhancing T Lymphocytes from Tumour-bearing Mice Suppress Host Resistance to a Syngeneic Tumour. *Eur. J. Immunol.*, **4**, 722.

UMIEL, T. & TRAINING, N. (1974) Immunological Enhancement of Tumour Growth by Syngeneic Thymus-derived Lymphocytes. *Transplantation*, **18**, 244.

WEXLER, H. (1966) Accurate Identification of Experimental Pulmonary Metastases. *J. natn. Cancer Inst.*, **36**, 641.

WOODRUFF, M. F. A. & BOAK, J. L. (1966) Inhibitory Effect of Injection of *Corynebacterium parvum* on the Growth of Tumour Transplants in Isogenic Hosts. *Br. J. Cancer*, **20**, 345.

WOODRUFF, M. F. A. & DUNBAR, N. (1975) Effect of Local Injection of *Corynebacterium parvum* on the Growth of a Murine Fibrosarcoma. *Br. J. Cancer*, **32**, 34.

WOODRUFF, M. F. A., DUNBAR, N. & GHAFFAR, A. (1973) The Growth of Tumours in T-cell Deprived Mice and their Response to Treatment with *Corynebacterium parvum*. *Proc. R. Soc., B.*, **184**, 97.